# Clotrimazole-Betamethasone Dipropionate Prescribing for Nonfungal Skin Conditions

**DOI:** 10.1001/jamanetworkopen.2024.11721

**Published:** 2024-05-16

**Authors:** Jeremy A. W. Gold, Avrom S. Caplan, Kaitlin Benedict, Shari R. Lipner, Dallas J. Smith

**Affiliations:** 1 Mycotic Diseases Branch, Centers for Disease Control and Prevention, Atlanta, Georgia; 2 Ronald O. Perelman Department of Dermatology, New York University Grossman School of Medicine, New York; 3 Department of Dermatology, Weill Cornell Medicine, New York, New York

## Abstract

This cross-sectional study identifies the common diagnoses and physician encounter types associated with clotrimazole-betamethasone dipropionate prescriptions among Medicare enrollees in 2021.

## Introduction

Visual inspection cannot reliably distinguish fungal rashes from nonfungal inflammatory skin conditions (eg, dermatitis, eczema).^1^ Misdiagnosis can lead to unnecessary antifungal use for nonfungal conditions or topical corticosteroid use for fungal rashes, potentially worsening infections.^2^ When rash causes are uncertain, physicians may prescribe topical antifungal-corticosteroid combination products, which are often less effective and more expensive than monotherapy for fungal skin infections^3,4^ and whose overuse is likely associated with the global emergence and spread of antimicrobial-resistant dermatophytosis.^2^

In the US, clotrimazole-betamethasone dipropionate is the most commonly prescribed topical antifungal-corticosteroid medication and is indicated to treat inflammatory dermatophytosis (ringworm).^3,4^ It is not recommended for use in persons younger than 17 years or intravaginally because betamethasone dipropionate is a high-potency corticosteroid. Recent clotrimazole-betamethasone use data in adults are sparse, but a single-center study including children and adults found only 38.1% of clotrimazole-betamethasone prescriptions were written for fungal diagnoses.^3^ We characterized clotrimazole-betamethasone prescribing in a large commercial health insurance database to identify potential physician knowledge gaps and improve patient treatment.

## Methods

We analyzed deidentified Merative MarketScan Commercial and Medicare Supplemental data to identify enrollees prescribed with clotrimazole-betamethasone during 2021. We estimated prescribing rates, stratifying by age group and other demographic characteristics. Among adults, we examined physician encounter types, antifungal prescriptions, and diagnostic testing and diagnoses with *Current Procedural Terminology* and *International Statistical Classification of Diseases and Related Health Problems, Tenth Revision* codes (eTable in Supplement 1). The Centers for Disease Control and Prevention Institutional Review Board deemed this study exempt from review because it was nonhuman participant research. We followed the STROBE reporting guideline. Analysis was performed between October and November 2023, using SAS, version 9.4 (SAS Institute Inc).

## Results

During 2021, 137 925 patients (83 580 females [60.6%], 54 345 males [39.4%]; mean [SD] age, 48.6 [18.0] years) were prescribed clotrimazole-betamethasone, 48.7% were from the US South (Table 1). Overall, the prescribing rate per 1000 enrollees was 6.7 (137 925 of 20 733 115), with higher rates among enrollees 18 years or older (8.1) vs younger than 18 years (1.3), females (7.6), and Northeast (8.8) and nonrural (6.7) residents.

**Table 1.** Clotrimazole-Betamethasone Dipropionate Prescribing Rates in a Large Cohort of Commercially Insured Patients in 2021

Characteristic	Patients prescribed with clotrimazole-betamethasone, No. (%)	No. of enrollees	Rate per 1000 enrollees
Age group, y			
<18	5558 (4.0)	4 356 166	1.3
<1	306 (0.2)	291 099	1.1
1-3	696 (0.5)	586 587	1.2
4-12	2032 (1.5)	2 084 976	1.0
13-17	2524 (1.8)	1 393 504	1.8
≥18	132 367 (96.0)	16 376 949	8.1
18-34	26 095 (18.9)	5 213 908	5.0
35-64	85 082 (61.7)	9 908 804	8.6
≥65	21 190 (15.4)	1 254 237	16.9
Sex			
Male	54 345 (39.4)	9 752 948	5.6
Female	83 580 (60.6)	10 980 167	7.6
US Census region of primary beneficiary’s residence			
Northeast	27 775 (20.1)	3 138 501	8.8
Midwest	29 049 (21.1)	4 684 556	6.2
South	67 185 (48.7)	9 275 076	7.2
West	13 236 (9.6)	3 572 033	3.7
Unknown	680 (0.5)	62 949	10.8
Urban or rural classification			
Nonrural	123 367 (89.4)	18 467 599	6.7
Rural	14 442 (10.5)	2 234 723	6.5
Unknown	116 (0.1)	30 793	3.8
Total	137 925 (100.0)	20 733 115	6.7

Among 113 227 adults prescribed with clotrimazole-betamethasone, family practice or internal medicine physician encounters were most common (40.7%), followed by obstetricians-gynecologists (13.6%) (Table 2). Few patients (3.5%) saw a dermatologist and few received topical antifungal monotherapy (1.1%), oral antifungals (2.6%), or diagnostic testing (15.0%). A minority of patients (35 321 [31.2%]) had a fungal diagnosis, most often dermatophytosis (63.5%) and candidiasis (33.5%). Fungal diagnosis was most frequent among enrollees who saw a podiatrist (77.0%); for each remaining physician encounter type, less than 45% of patients received a fungal diagnosis. Among enrollees without a fungal diagnosis (77 956), common diagnoses were dermatitis and eczema (22.6%), nonfungal genital conditions (13.4%), and rash and other nonspecific skin eruption (12.0%).

**Table 2.** Encounter Type, Antifungal Treatments, Testing, and Diagnoses for Adults Prescribed With Clotrimazole-Betamethasone Dipropionate in 2021 (N = 113 227)^a^

Characteristic	No. (%)
Encounter type	
Family practice or internal medicine	46 070 (40.7)
Obstetrician-gynecologist	15 421 (13.6)
Acute care hospital	14 488 (12.8)
Nurse practitioner or physician assistant	12 545 (11.1)
Urgent care or emergency medicine	4793 (4.2)
Podiatry	4733 (4.2)
Dermatology	3977 (3.5)
Urology	2560 (2.3)
Otolaryngology	2514 (2.2)
None of the above^b^	31 262 (27.6)
Topical antifungal monotherapy	1297 (1.1)
Oral antifungals	2962 (2.6)
Diagnostic testing	16 989 (15.0)
Fungal culture	1132 (1.0)
Direct microscopy	4142 (3.7)
Susceptibility testing	4896 (4.3)
Skin biopsy	4515 (4.0)
Polymerase chain reaction	5134 (4.5)
Fungal diagnosis present^c^	35 321 (31.2)
Dermatophytosis	22 426 (63.5)
Candidiasis	11 844 (33.5)
Other superficial mycoses	2487 (7.0)
Unspecified mycoses	275 (0.8)
Fungal diagnosis absent	77 956 (68.8)
Dermatitis and eczema	17 607 (22.6)
Genital conditions	10 422 (13.4)
Acute vaginitis	5091 (48.8)
Rash and other nonspecific skin eruption	9345 (12.0)
Other local infections of skin and subcutaneous tissue	4077 (5.2)

^a^ Data include adults (aged ≥18 years) with continuous insurance enrollment during the 90 days before to 90 days after initial clotrimazole-betamethasone prescription (85.6% of all adults prescribed clotrimazole-betamethasone in 2021). Encounter type includes encounters occurring in the 7 days before to 7 days after the initial clotrimazole-betamethasone prescription. Topical antifungal monotherapy and oral antifungals include those prescribed during the 14 days to 1 day before initial clotrimazole-betamethasone prescription. Diagnoses and diagnostic testing include those documented in the 90 days before to 90 days after the first clotrimazole-betamethasone prescription. Patients could have had more than 1 encounter type, antifungal treatment, diagnosis, and test type.

^b^ The most commonly listed other encounter types overall were laboratory (n = 3893), other facility (n = 3163), and multispecialty physician group (n = 1142).

^c^ Overall, the frequency of fungal diagnosis receipt was highest for patients who saw a podiatrist (77.0%); for each remaining encounter type, less than 45% of patients received a fungal diagnosis.

## Discussion

Less than one-third of adults prescribed with clotrimazole-betamethasone received a fungal diagnosis, with the remainder of patients receiving diagnoses for noninfectious dermatitis, eczema, nonfungal genital conditions, and nonspecific rashes, similar to a study of clotrimazole-betamethasone prescribing in children.^4^ Consistent with prior studies, clotrimazole-betamethasone prescriptions were more frequently associated with nondermatologist visits,^3,4^ potentially reflecting the lack of awareness that it contains a high-potency corticosteroid and poses potential harms associated with indiscriminate use, including adverse effects and resistance selection pressure.^2,4^ Observed prescribing for patients younger than 17 years and patients diagnosed with vaginitis suggests inappropriate prescribing and opportunities for physician education.^3,4^ Fungal testing was infrequent, consistent with a prior study^5^ and suggesting opportunities to increase diagnostic accuracy.^1^

Study limitations include potential misclassification inherent to claims data. Furthermore, clotrimazole-betamethasone prescribing practices might differ among patients with noncommercial insurance.

Instead of prescribing clotrimazole-betamethasone empirically, physicians can confirm fungal diagnoses using potassium hydroxide with microscopy, fungal culture, or DNA-based techniques^6^ or can refer patients to a dermatologist. For confirmed fungal skin infections, instead of prescribing clotrimazole-betamethasone, physicians may consider prescribing antifungal monotherapy, which can help decrease inflammation and associated pruritus.

## Notes

Supplement 1. **eTable.** International Classification of Diseases, Tenth Revision, Clinical Modification (ICD-10-CM) and Current Procedural Terminology (CPT) Codes Used to Identify Features of Interest

Supplement 2.
Data Sharing Statement
