# Supplementary material for: Clotrimazole-Betamethasone Dipropionate Prescribing for Nonfungal Skin Conditions
Source: JAMA Netw Open. 2024 May 16;7(5):e2411721. doi: 10.1001/jamanetworkopen.2024.11721 (PMC11099679; doi:10.1001/jamanetworkopen.2024.11721)
Supplement: Supplement 1. — eTable. International Classification of Diseases, Tenth Revision, Clinical Modification (ICD-10-CM) and Current Procedural Terminology (CPT) Codes Used to Identify Features of Interest [file jamanetwopen-e2411721-s001.pdf]

# Supplemental Online Content

Gold JAW, Caplan AS, Benedict K, Lipner SR, Smith DJ. Clotrimazole-betamethasone dipropionate prescribing for nonfungal skin conditions. *JAMA Netw Open*. 2024;7(5):e2411721. doi:10.1001/jamanetworkopen.2024.11721

**eTable.** International Classification of Diseases, Tenth Revision, Clinical Modification (ICD-10-CM) and Current Procedural Terminology (CPT) Codes Used to Identify Features of Interest

This supplemental material has been provided by the authors to give readers additional information about their work.

© 2024 Gold JAW et al. *JAMA Network Open*

**eTable. International Classification of Diseases, Tenth Revision, Clinical Modification (ICD-10-CM) and Current Procedural Terminology (CPT) codes used to identify features of interest**

| | |
|---|---|
| **ICD-10 Codes** | |
| Fungal diagnoses | |
| Dermatophytosis | B35 |
| Other superficial mycoses | B36 |
| Candidiasis | B37 |
| Unspecified mycoses | B49 |
| Non-fungal diagnoses | |
| Infections of the skin and subcutaneous tissue (non-fungal) | L00–L08 |
| Dermatitis and eczema | L20–L30 |
| Genital conditions | |
| Other disorders of prepuce | N47.8 |
| Balanitis | N48.1 |
| Disorder of penis, unspecified | N48.9 |
| Acute vaginitis | N76.0 |
| Acute vulvitis | N76.2 |
| Other specified noninflammatory disorders of vagina | N89.8 |
| Rash and other nonspecific skin eruption | R21 |
| **CPT codes** | |
| Diagnostic testing | |
| Fungal culture | 87101, 87102, 87106, 87107 |
| Direct microscopy | 87210, 87220, 87206 |
| Susceptibility testing | 87186 |
| Skin biopsy | 11100, 11102, 11103, 11104, 11105, 11106, 11107 |
| Polymerase chain reaction | 87481, 87798, 87800, 87801 |

© 2024 Gold JAW et al. *JAMA Network Open*
