# Supplementary material for: Clotrimazole-Betamethasone Dipropionate Prescribing for Nonfungal Skin Conditions
Source: JAMA Netw Open. 2024 May 16;7(5):e2411721. doi: 10.1001/jamanetworkopen.2024.11721 (PMC11099679; doi:10.1001/jamanetworkopen.2024.11721)
Supplement: Supplement 2. — Data Sharing Statement [file jamanetwopen-e2411721-s002.pdf]

Data Sharing Statement

Gold. Clotrimazole-Betamethasone Dipropionate Prescribing for Nonfungal Skin Conditions. *JAMA Netw Open*. Published May 16, 2024. doi:10.1001/jamanetworkopen.2024.11721

Data
Data available: No

Additional Information
Explanation for why data not available: This study used third-party data that we cannot legally distribute. All relevant summary data are within the manuscript and the supporting files. The raw data underlying the results presented are available from the Merative Marketscan Research Databases: https://www.merative.com/documents/brief/marketscan-explainer-general. Others can access the data by going to this website and contacting Merative. The authors did not have any special access privileges that others would not have.
